# High-fidelity simulation in healthcare education: Design and delivery considerations for optimising teaching and learning in higher education

**DOI:** 10.29045/14784726.2025.9.10.2.55

**Published:** 2025-09-01

**Authors:** Jon Newton

**Affiliations:** University of the West of England ORCID iD: https://orcid.org/0000-0001-5407-0694

**Keywords:** graduate education, high-fidelity simulation training, nurse, paramedic, physician, problem-based learning

## Abstract

**Introduction:**

Simulation-based learning (SBL) is a recognised teaching and learning tool within higher education (HE) and one capable of facilitating skill retention and knowledge retrieval. Successfully achieving these outcomes relies on effective design, delivery and debriefing; yet a limited range of publications draw together these fundamental components. High-fidelity simulation (HFS) describes a sub-division of SBL that, in recent years, has generated traction within healthcare education.

**Aims:**

To support educators in orchestrating HFS with greater impact and influence, the author set out to compose an article outlining five constructs that collectively possess scope to optimise HE teaching and learning outcomes. These five constructs consist of: (1) creating a believable scenario; (2) integrating the five principles of ‘fidelity harmony’; (3) selecting an appropriate modality; (4) adopting a clear pedagogical stance; and (5) amalgamating concepts of experiential learning theory into the briefing and debriefing. When dynamically incorporated, important gaps between theory and practice can be bridged and learner experience will be significantly enhanced.

**Conclusion:**

This article offers HE educators a series of recommendations for creating deeply immersive learning experiences for augmenting learner performance, and provides a new definition for HFS, which challenges the erroneous notion that ‘high fidelity’ represents ‘high technology’.

## Introduction

Simulation-based learning (SBL) is a recognised teaching and learning tool in higher education (HE) and one capable of facilitating skill retention and knowledge retrieval (Carey & Rossler, 2022; El Hussein & Hirst, 2023; Watson et al., 2021). If we consider SBL as an umbrella term, under which sit three categories – ‘low-fidelity’, ‘mid-fidelity’ and ‘high-fidelity’ – educational activities and SBL planning can be further focused (Kim et al., 2016). These three categories represent a scale through which environmental realism and scenario believability are portrayed, although these categories may be counter-intuitively considered hierarchical in nature. However, this is inaccurate, and one category should not be considered *better* than another (Carey & Rossler, 2022). Fidelity exists on a spectrum, but ‘more’ is not necessarily preferable. Instead, the level of realism created by educators should align with the ability of the learner and the intended learning objectives (Goodwin & Nestel, 2024).

The term high-fidelity simulation (HFS) represents a multi-dimensional concept, corresponding to the degree of realism created through the selection of equipment, setting and scenario (Berro et al., 2023; Dieckmann et al., 2007). This article sets out five HFS considerations for educators to contemplate, and how these can be tuned to optimise learning outcomes and improve student experience.

## Background

SBL has scope to cultivate *cognitive apprentices* – a theory that defines how educators support and stretch students to manage complex intellectual tasks in a life-like (rather than theoretical) manner (Culatta, 2020; Spector, 2008). Traditional or didactic lectures and assignment writing may serve to instil background knowledge, but seldom do they provide learners with the practical skills necessary for real-world practice encounters. HFS provides a platform for kinaesthetic, heuristic learning; and while educators instinctively recognise these benefits, shortcomings in pedagogical knowledge and simulation aptitude can prevent effective translation (Altabbaa et al., 2019). Inadequately considered scenarios, sub-optimal planning and ineffectively executed simulations lead to student frustration, professional dissatisfaction and damaged team dynamics and can crucially prevent learning outcomes being achieved (Bearman et al., 2018; Kable et al., 2012; Tosterud, 2015).

The first step for designing a healthcare simulation is to define the learning goal (Harrington & Simon, 2022). Recognising this is rarely the difficulty; instead, it is understanding how best to accomplish it. ‘Simulation’ and ‘clinical skills’ are terms often grouped together in the healthcare world, but a common misconception held by students, clinicians and educators alike is that they are the same thing. This incorrect assumption can lead to communication challenges when briefing colleagues regarding the anticipated learning approach and method of delivery. The acquisition of appropriate theoretical knowledge must first be instilled, followed by opportunities to practise the clinical skill/s within the safety of a classroom-based setting. The last step should be to integrate the clinical skill/s within a simulation. It is important to appreciate that any clinical skill should first be honed before attempting to evaluate a learner’s ability to recognise the need for that skill and their ability to perform it under the inherent pressure of a simulated scenario.

HFS has unique facets, but it is no different from any other teaching and learning environment; which requires an analysis of learning needs, student goals and the development/delivery of an educational system that meets its requirements (Fry et al., 2014). HFS has evolved into a complex science over the past 20 years and is perhaps the most challenging of simulation-based-learning activities for institutions to undertake because it is almost always time-consuming and costly, and even the highest quality exercises possess significant limitations (Elendu et al., 2024; Hanshaw & Dickerson, 2020; Maloney & Haines, 2016; Newton & Smith, 2024). To mitigate for the limitations in any SBL event, especially when attempting to deliver HFS, the following five considerations may be useful.

### 1. Scenario

As a rule, the scenario chosen for HFS should always be representative of a plausible, real-world practice encounter and be designed to play out in such a way that it is believable to learners and observers (Howard, 2018). If a scenario tries to do too much, or lacks authenticity, concentration may wain and educational gains will not be optimised. Sympathetically crafting scenarios influenced by real-world events and experiences from an educator’s own practice can manifest as a powerful teaching and learning tool. This is because it promotes the integration of subtleties within a scenario that help suspend learner disbelief, facilitate cognitive buy-in and enable learners to behave as if the simulation were in fact real (Lavoie et al., 2020).

### 2. Fidelity

Pedagogical stance, instructor experience and resource availability are major influencers in simulation design, development and delivery, but at any level, effective simulation planning relies on the selection of the most appropriate level of ‘fidelity’ (Chiniara et al., 2013). If we accept the notion that low-fidelity simulation builds knowledge, mid-fidelity develops competence and high-fidelity augments performance, then teaching and learning objectives can be more effectively scaffolded (Carey & Rossler, 2022). HFS should be avoided when learners lack knowledge and competence, as this is likely to negatively impact confidence, engagement and progression. Mid-fidelity simulation is best delivered to trainees who possess knowledge but lack competence; and lastly, low fidelity helps instil knowledge in a practical way. The recipe for a successful simulation is to first build knowledge, then competence and finally performance.

Once an educator has devised a scenario and selected an appropriate level of fidelity (low, mid or high), the next step should be to further scaffold the simulation design by considering the five sub-categories of fidelity – physical, conceptual, psychological, functional and sociological – and then amalgamating a blend of these into the scenario (Burford et al., 2023; Lavoie et al., 2020; Tun et al., 2015). These five sub-categories correspond with a different aspect of authenticity within a simulation and represent a control measure for preventing learning impedance (Norman et al., 2012).

Physical fidelity is the depth to which a simulated environment reflects what a participant sees, hears, feels or smells (Gajrani, 2021). Conceptual fidelity is the depth of scripting and planning of the scenario so that it accurately reflects the way the scene would present in real-world practice (Rudolph et al., 2007). Psychological fidelity is the emotional responses and behaviours elicited by participants as if the simulation were in fact real (Adamson, 2015; Munshi et al., 2015). These three types of fidelity can complement and overlap, yet they can also detract if not effectively balanced (Naismith et al., 2020). To help contextualise this notion, an educator could heighten the physical fidelity of a scenario by adding realistic background noises to a scene (such as a crowd of people shouting or screaming); however, this stimulus might raise learner stress levels beyond their threshold and hinder learning. Intuitively, we may think that the more realistic an environment, the more it will enhance learning, but without having first provided the learner with the requisite skillset to navigate specific stimuli, exposure can in fact serve as an unhelpful obstacle (Choi et al., 2017).

Obtaining satisfactory harmony between these fidelities mentally signposts learners into a space where *real* learning can occur. This will typically be outside their comfort zone, but not far enough to harm confidence, clinical ability or well-being. When fidelity is robustly considered, participants can suspend disbelief and accept the simulation as if it were real (Adamson, 2015; Dieckmann et al., 2007; Masotta et al., 2021; Watts et al., 2021). Creating exercises with sufficient depth to reach this disposition is routinely challenging, but lapses in physical fidelity are preferable to lapses in conceptual fidelity (Smink et al., 2017). Learners need a scenario that makes sense, and if educators deliver something which feels true to life, participants are more able to accept the artificial aspects of the physical environment (Dieckmann et al., 2007). The common denominator for lapses in physical fidelity (particularly in HE) will typically be the fact that a classroom or skills lab is being used, which will inherently lead to losses in environmental realism. However, if the scenario feels genuine, learners can apply knowledge and problem-solving skills through a heuristic and experimental approach. Recruiting subject matter experts to help develop/review the proposed scenario and perform pilot tests represents best practice for securing conceptual fidelity (Smink et al., 2017). It is also beneficial to deliver simulation as an integrated component of standard curriculum, instead of as an extraordinary event (Motola et al., 2013).

In larger exercises or an interprofessional HFS, functional fidelity and sociological fidelity are particularly important. Functional fidelity refers to the dynamic interaction between participants and the assigned task, which is essential when teaching technical or psychomotor skills (Carey & Rossler, 2022; Choi et al., 2017). The more precise the skill or procedure being performed, the higher the level of functional fidelity required (Choi & Wong, 2019). Sociological fidelity relates to interdisciplinary simulation and corresponds directly with the level of interactive realism between separate groups of learners within a simulation (Armenia et al., 2018; Thomas & Reeves, 2015). For example, if a road traffic collision were being simulated, paramedics, fire-fighters and police officers would need to interact at the scene. For the associated subtleties of these interactions to be authentically delivered, heightening the level of sociological fidelity would likely require input from educators attached to all three professional disciplines.

Careful consideration of learner needs, method of assessment and the intended endpoint can help enable delivery of quality outputs which are safe, enjoyable and in keeping with the desired learning objectives (Turner & Harder, 2018). Therefore, when considering the level of fidelity to utilise, *more* is not necessarily better, because learning is not proportionate with the level of realism provided (Carey & Rossler, 2022).

### 3. Modality

Another important consideration for HFS is the selection of a suitable modality. This term refers to the method/s, equipment and technology utilised. An array of products capable of demonstrating varied degrees of realism exist, and the selection of these tools should be guided by the type of simulated activity and learning outcomes desired (Carey & Rossler, 2022). Modality typically falls into four categories: (1) live play; (2) virtual reality; (3) augmented reality; and (4) mixed reality, although a simulation could be constructed using a combination of more than one modality.

Live-play simulation (LPS) is perhaps the most traditional of these approaches and is routinely utilised by the emergency services. LPS is typically delivered by constructing a mock emergency environment in either a real or simulated high-profile location (such as an airport, a shopping centre or a university). When ‘higher risk’ exercising is required, specialist training facilities are normally utilised. LPS is often supported using standardised patients (SPs), moulage and special-effects technology (e.g. smoke, fire and controlled explosions). LPS is often the modality of choice for the military and the emergency services, to prepare specialist and non-specialist assets to respond to critical or major incidents. LPS aims to promote critical thinking and practise the recognition/application of core skills and competencies. However, this type of exercising seldom represents HFS because it can contain: (a) ‘pause-and-reflect’ moments; (b) intermittent interruption of assigned character roles; (c) phrases such as ‘*ok, just pretend that* …’; and (d) the fast-forwarding of time, all of which prevent key skills, interventions or clinical competencies being authentically performed. Such actions are counterproductive because scenario realism will be lost. This causes cognitive shifting among participants, which diminishes the potential for honing critical thinking and the muscle memory associated with performing time critical skills. HFS should always play out in real time and encourage participants to autonomously find solutions in environments that possess uninterrupted realism and clinical authenticity.

Virtual reality (VR) simulation depicts the use of computer modelling and replication that enables interaction with an artificial, three-dimensional, visual or sensory environment (Nassar et al., 2021). This is typically achieved by the wearing of VR goggles, headsets, gloves or a body suit. This equipment enables the wearer to tour simulated environments, experiencing changing viewpoints and perspectives, which convincingly alter as the user turns their head or makes physical steps in a selected direction. This modality yields great promise; however, empirical evidence on VR is currently lacking because this technology is so recent and has not yet been broadly implemented in HE settings (Chernikova, 2020). VR also requires careful investment to ensure fit-for-purpose solutions are obtained. Bespoke packages will almost always be required to meet the needs of learners, and this will often be costly, time-consuming to build and require the involvement of specialist staff. Nevertheless, VR can offer institutions a sustainable and comparatively longer-term learning resource.

Augmented reality (AR) represents the technology we associate with ‘first-person shooter video games’ and the heads-up displays projected in vehicle cockpits and viewfinders (Zhou et al., 2024). AR systems have advanced significantly in recent years and transitioned into HE, enabling learners to interact with real-world environments, enhanced with virtual imagery (Al-Ansi et al., 2023). For example, computer-generated objects can be superimposed to co-exist with those in the real world, which effectively provides unique educational experiences in a safe space, whilst addressing and challenging the development of specific skillsets (González Almaguer, 2023).

Finally, mixed reality (MR) more comprehensively blends the physical and virtual world together (Flavián et al., 2019). MR shares similarities with AR, because both technologies integrate real-world objects alongside computer-generated ones, but MR allows the user to directly interact with them. Computer-generated and real-world objects can co-exist within a scenario, enabling individuals to engage more dynamically because a scenario could, for example, incorporate a mixture of both real and digitally created patients, environments and artefacts. MR therefore provides significant scope for teaching and learning innovation, although this approach may bias a learner’s emotional state and cognitive load. Moreover, the anxiety and stress perceived by students during the simulations could differ from real-world clinical situations and, thus, this modality should not be considered a replacement for real-world environment exercising (Brunzini et al., 2022).

Many HE institutions are beginning to incorporate VR, AR and MR; however, the need to offer parity of experience to large cohorts of students makes successful and consistent incorporation challenging. Restricted spaces to safely utilise this equipment can also impact successful integration. Nevertheless, this range of modalities provides educators with unrivalled opportunities for innovation and immersive learning in scenarios that would previously have been considered too unsafe and/or costly to contemplate delivering as LPS.

### 4. Pedagogy

Pedagogy is commonly defined as the art, science and craft of teaching and the ways in which learning objectives are achieved; formulated by teaching beliefs and the interplay between cultural, social, political and psychological influences (Shah, 2021). It has been long debated whether simulation represents a pedagogy in itself, and while the literature demonstrates conflicting viewpoints on this, there is a compelling argument to suggest not (Erlam et al., 2017). Instead, simulation is perhaps best described as a method of teaching and learning, influenced by the pedagogical building blocks of behaviourism, constructivism and cognitivism. Like all other teaching and learning methods, effective HFS design and delivery requires a sound understanding of educational learning theory and pedagogical practice. However, a sparsity of current research signals limited integration of these educational theories, especially in relation to HFS (Ross, 2021). Nevertheless, educators need to be credible in their approach and identify key pieces of literature that support and guide their teaching practices. There is also the need for educators to align pedagogical influences with their own morals and values to authentically hone their teaching craft.

### 5. Experiential learning theory

**Figure 1:**
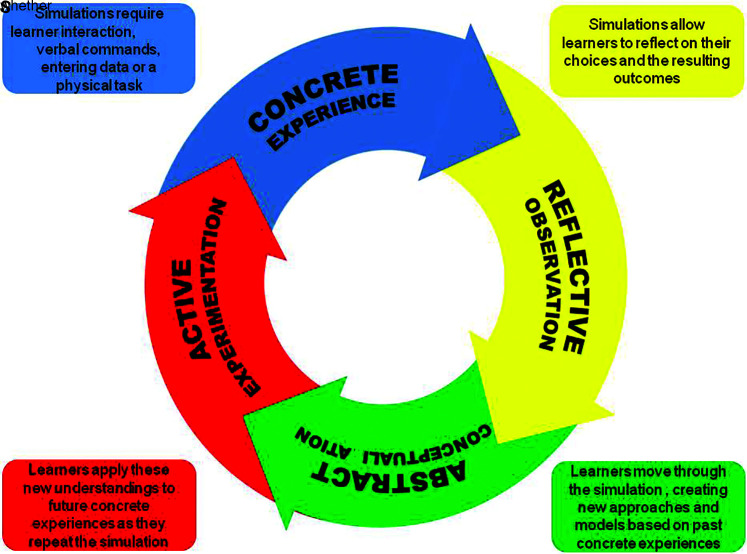
Figure 1. Kolb’s experiential learning cycle.

Experiential learning theory (ELT) was established in the 1980s by David Kolb and is synonymous with many constructivist concepts (McLeod, 2024). Kolb’s ‘experiential learning cycle’ represents a dynamic view of learning, portraying a cyclical process driven by the resolution of the dual dialectics of action/reflection and experience/abstraction (Kolb & Kolb, 2012). The experiential learning cycle is highly regarded in academia; and is perhaps the most scholarly influential and cited model in ELT (Morris, 2020). Figure 1 showcases an adapted version for SBL.

Kolb suggests that learning occurs through the transformation of experience in four distinct phases: (1) a concrete experience; (2) a reflective observation; (3) an abstract conceptualisation; and (4) active experimentation (Preceptor Education Program, 2015). Each phase feeds into the next and is mutually supportive. Learning from a single experience does not occur at one specific phase in the cycle and requires the entire cycle to be effective. Learning over time does not occur in individual cycles either, but as a spiral, with each concrete experience and cycle building off from those before it. Education must therefore be conceived as a continuing reconstruction of experience, and seminal works showcase that the ‘process’ and ‘goal’ of education should be considered as one and the same thing (Dewey, 1897).

HFS should also include effective pre-briefing, briefing and debriefing (Tong et al., 2022). The complexity and immersive realism inherent in HFS require careful navigation because the emotional responses invoked by learners can closely resemble those experienced in real-world practice, even though no actual harm to people, wildlife, property or possessions has occurred. Pedagogical influences should guide HE educators in their approach to managing these encounters, and while a variety of tools exist to debrief learners following SBL activities, little consensus exists to support the use of a specific model or approach. Some tools may also not extrapolate well to large-scale exercises, interprofessional working or activities simultaneously spanning multiple geographical environments.

Non-simulation-based learning (NSBL) is widely utilised to teach theoretical concepts to learners, using didactic lecture-based training and problem-based learning (Mejia et al., 2018). NSBL represents a respected teaching and learning tool for technical knowledge acquisition; however, as a standalone approach it typically fails to equip learners with the requisite skillset needed for handling ‘critical events’ and developing teamworking abilities because it does not offer an authentic ‘in the moment’ learning experience (Su & Zeng, 2023). Nevertheless, NSBL has a key role within the broader context of attaining knowledge and skills within HE settings. It is perhaps better situated within the early stage of a module, as part of a scaffolded learning journey. When practicable and appropriate, concluding modules with an HFS can be instrumental in augmenting learner performance. Case Study 1 (below) provides an illustrative example.

### Case study 1

In 2021, the author introduced a novel approach to paramedic education by using a ‘technical/tactical/life-like’ (TTL) teaching framework. The framework was devised to more effectively scaffold a learning journey within a 15-credit module at Level 6 titled ‘Major Incident Clinical Care’. The ‘technical’ element of the curriculum was covered by using NSBL. The ‘tactical’ aspect was delivered via an interprofessional major incident table-top exercise, utilising a mid-fidelity simulation methodology to develop learner competencies in critical thinking, problem solving and teamwork. Finally, the ‘life-like’ component was accomplished through an interprofessional HFS to augment learner performance. The TTL framework incorporated the NHS England Emergency Preparedness Resilience and Response (EPRR) guidelines and was presented at the Association for Simulated Practice in Healthcare (ASPiH) conference at Brighton Metropole in 2023. The TTL approach has since been embedded within a further curriculum at the author’s HE institution and the module’s accompanying HFSs have since been recognised at an international level for teaching excellence; several of which represent the largest interprofessional major incident simulations conducted to date within the UK (Newton & Smith, 2024).

### Pre-simulation briefing

Psychological safety is an underpinning philosophy for creating simulation experiences in which learners can develop within a stimulating and challenging, yet supportive social atmosphere (Somerville et al., 2023). Simulation pre-briefing should not be rushed or overlooked by educators, and it should be utilised to effectively share intended learning objectives and prepare learners for the task ahead (Hughes & Hughes, 2023). When robustly achieved, this reduces anxiety, promotes psychological safety and will enhance the learning experience. The 12 tips as recommended by Somerville et al. (2023) for conducting a pre-brief enable educators to promote a psychologically safe environment. The optimal balance is perhaps best evidenced when educators reach a stage whereby learners can fully ‘buy into’ the SBL experience to such an extent that they treat the scenario as if it were real; whilst feeling empowered to make mistakes without fear of judgement or consequence. It is important to recognise that HFS can be stressful and promote feelings of discomfort, and while this can be beneficial in promoting learner gains, the absence of a well-conducted pre-simulation briefing can negatively impact learning (Newton & Smith, 2024). Clarity and transparency in this aspect of simulation design and delivery helps to ensure that learners are suitably prepared, thereby enhancing psychological safety (Somerville et al., 2023).

### Post-simulation debriefing

This final element is fundamental to the learning process due to its links with promoting reflective practices and experiential learning theory (Abulebda et al., 2023; Dieckmann et al., 2009; Fanning & Gaba, 2007). Debriefing provides not only a valuable opportunity to reinforce psychological safety but also extended scope to address clinical questions and any developing anxieties within a group of learners. Within the context of interprofessional major incident simulation, post-simulation debriefing is underdeveloped. The author presents a novel three-step approach to interprofessional simulation debriefing in a subsequent manuscript, titled ‘The Trinity Technique: A novel 3-step approach for debriefing interprofessional major incident simulation’ (Newton, 2025). Therefore, this particular element is not explored further within this article.

## Discussion

SBL generally, and HFS particularly, can subject learners to higher levels of cognitive load than traditional learning and teaching approaches. However, if we accept that the ‘ultimate goal’ is to properly equip learners to deal with the realities of real-world practice, we can more effectively scaffold a learning journey. Attitudes, values and behaviours may also be shaped by HFS, fostering a deeper appreciation of the experiential learning cycle as a lifelong learning strategy (Armenia et al., 2018; Duphily, 2014).

**Figure 2:**
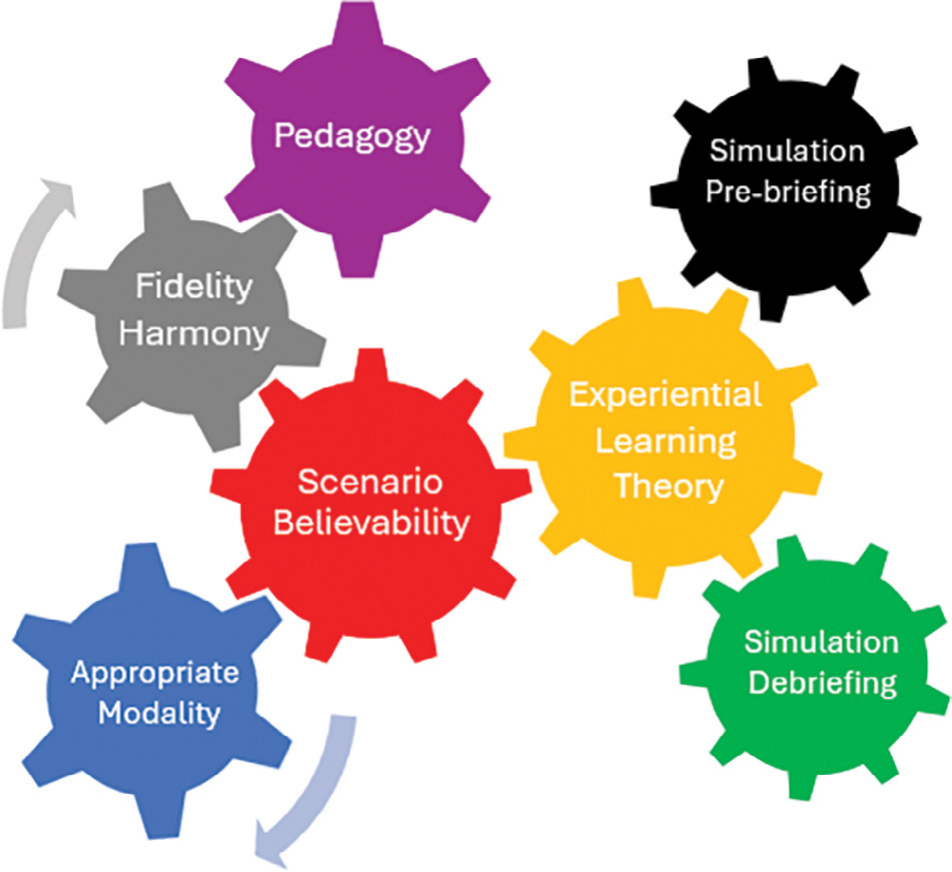
Figure 2. High-fidelity simulation design and delivery ‘best-practice’ recommendations.

The construction of any simulation and its desired learning objectives will inevitably be unique to its developers, but perhaps the most important consideration in the whole process is learner well-being. It is important to appreciate that if the cognitive burden is too heavy, this will lead to overload and ineffective learning (Carey & Rossler, 2022). Implementing reliable and structured safety-netting mechanisms will allow shortfalls to be detected and rectified. HFS can subject learners to much higher levels of extraneous cognitive load than intended; thus, adopting methods capable of nurturing learner development is a worthy consideration. The evolution of simulation in healthcare education has undergone such advancement that it can be considered parallel with clinical practice experience in terms of skills, knowledge and confidence acquisition (Roberts et al., 2019). Despite this, it should not replace real-world practice learning, because these environments are (quite rightly) restricted to ensure that clinical need and patient safety remain the priority. These settings therefore cannot provide a ‘safe space’ for learners to make mistakes without consequence, which is why simulation represents such a valuable teaching and learning commodity. Figure 2 summarises the five fundamental concepts discussed in this article as a ‘best-practice’ infographic.

### The five fundamentals of HFS

- Create a realistic and believable scenario to which learners and educators can properly ‘buy in’.
- Ensure ‘fidelity harmony’ by balancing the five fidelities.
- Select the most appropriate modality for the intended learning objective/s.
- Adopt a clear pedagogical stance to improve focus and teaching authenticity.
- Integrate experiential learning theory into the pre-simulation briefing and post-simulation debriefing.

### Limitations

This professional practice article draws together foundational knowledge and phenomena to aid educational developments and promote future research undertakings. The article was composed from a combination of influential studies, the author’s experience within this field of practice and industry observation, but no formal research was undertaken to support the best-practice recommendations outlined herein. Nevertheless, this article provides a useful contribution to the literature within this specialist area of healthcare education by amalgamating crucial aspects of HFS design and delivery to aid HE educators in improving teaching and learning practices.

## Conclusion

HFS offers unrivalled opportunities for teaching and learning innovation. When effectively co-ordinated, important gaps between theory and practice can be bridged. This position may be further optimised when educators dynamically integrate the best-practice recommendations outlined in Figure 2. When effectively realised, this will produce deeply immersive learning experiences capable of augmenting learner performance. This synthesis of the wider literature, coupled with the author’s experiences, provides opportunities for revised thinking and an alternative definition for HFS, which is representative of fine-tuning five principles to achieve a ‘fidelity harmony’. In addition, the notion that high fidelity equates with ‘high technology’ should be challenged.

Implementing sustainable simulation programmes takes time, expertise and financial commitment, but even well-conducted one-off exercises can produce valuable learning experiences and generate data capable of addressing vital research-knowledge gaps. Further research is now required to expand the literature within this domain to alleviate the current lack of high-quality randomised studies that evaluate the influence of HFS design and delivery, and its impact on actual patient outcomes.

## Guarantor statement

JN designed, wrote and produced the manuscript. JN acts as the guarantor for this article.

## Conflict of interest

None declared.

## Ethics

All methods were carried out in accordance with the Declaration of Helsinki, as well as all other relevant guidelines and regulations.

## Funding

None.

## Statement of generative AI in scientific writing

The author did not use a generative artificial intelligence (AI) tool or service to assist with preparation or editing of this work.
